# The Treatment of Keloid Scars via Modulating Heterogeneous Gelatin-Structured Composite Microneedles to Control Transdermal Dual-Drug Release

**DOI:** 10.3390/polym14204436

**Published:** 2022-10-20

**Authors:** Yong-Ji Chen, Hung-Wei Cheng, Wan-Yu Yen, Jen-Hao Tsai, Chin-Yi Yeh, Ching-Jung Chen, Jen Tsai Liu, San-Yuan Chen, Shwu-Jen Chang

**Affiliations:** 1 Department of Biomedical Engineering, I-Shou University, Kaohsiung 82445, Taiwan; 2 Department of Materials Science and Engineering, National Yang Ming Chiao Tung University, Hsinchu 30010, Taiwan; 3 Research Center for Materials Science and Opti-Electronic Technology, School of Opto-Electronic Technology, University of Chinese Academy of Sciences, Beijing 100049, China; 4 Research Center for Materials Science and Opti-Electronic Technology, College of Materials Science and Opto-Electronic Technology, University of Chinese Academy of Sciences, Beijing 100049, China; 5 Graduate Institute of Biomedical Science, China Medical University, Taichung 406040, Taiwan; 6 School of Dentistry, College of Dental Medicine, Kaohsiung Medical University, Kaohsiung 80708, Taiwan

**Keywords:** keloid scar, gallic acid, quercetin, hydrogel microneedle, nanocarrier

## Abstract

Keloid scarring is an abnormal scar disease characterised by excessive proliferation of fibroblasts and over-deposition of collagen during wound healing. Although various treatments for keloid scars have been developed, preventive medicine is believed to be a promising strategy. The skin barrier limits the gentle topical administration of medicaments such as creams and hydrogel dressings, resulting in reduced therapeutic efficacy. In recent years, microneedles (MNs) have been regarded as an appreciable device for topical administration without inducing side effects, and they are painless and do not cause bleeding. In this study, an MN patch with controlled transdermal dual-drug release was developed to achieve combinatory treatment of keloid scars using a heterogeneous gelatin-structured composite MN. Gelatin hydrogel was used as a substrate to load gallic acid (GA) and quercetin-loaded amphiphilic gelatin nanoparticles to fabricate dual-drug heterogeneous composite MNs. The results of the insertion test and mechanical properties of the MNs showed that the heterogeneous composite MN patches could be self-pressed into the stratum corneum and control dual-drug release at different time periods. GA was released at an earlier stage to retard the proliferation of fibroblasts, and quercetin was released at a later stage as a strong antioxidant to erase the generation of reactive oxygen species. Furthermore, real-time quantitative polymerase chain reaction data indicated that the gene expression of fibroblasts (such as Col I and III) was downregulated in the dual-drug system. The above results demonstrate that using heterogeneous composite MNs with the combination of dual-drug pharmacology is beneficial for preventing keloid scar formation.

## 1. Introduction

Keloid scars are abnormal tissue scars that occur during wound healing. During the proliferation stage of skin wound healing [1], some fibroblasts gradually differentiate into myofibroblasts to promote shrinkage of the wound edges [2,3]. However, if fibroblasts are excessively activated during wound healing, excessive collagen can be secreted, resulting in an imbalance between the synthesis and degradation of the extracellular matrix (ECM), which leads to the formation of keloid scars [4,5]. To date, various clinical therapeutic strategies have been developed for the treatment of keloid scars, including prophylactic treatment, surgical excision, laser therapy, and intralesional corticosteroid injection [6,7,8]. Compared with other therapeutic strategies, prevention of pathological keloid scarring can help avoid unnecessary scarring; thus, prophylactic treatment is considered superior to other treatments.

In recent years, natural compounds have been used to prevent the formation of keloid scars due to the proliferation of fibroblasts. Among them, flavonoids and phenolic acid compounds, such as gallic acid (GA), caffeic acid, and ferulic acid, are widespread in nature [9], and they have been reported to have antioxidant, anti-inflammatory, and antimicrobial properties. GA is a phenolic acid that efficiently prevents wound fibrosis owing to its strong free-radical scavenging ability. Moreover, GA can regulate collagen secretion to improve re-epithelisation in chronic wounds [10,11]. Therefore, GA is considered a promising candidate for keloid prophylaxis. In addition to phenolic acid compounds, flavonoid compounds, such as quercetin (Qu), kaempferol, and curcumin, have been used for their antioxidant, anti-inflammatory, and anticarcinogenic properties [12,13]. More importantly, flavonoid compounds can selectively bind with the type I receptor of transforming growth factor beta (TGF-β) to reduce collagen hypersecretion, which avoids keloid formation [14]. Among them, Qu has been demonstrated to regulate the proliferation of fibroblasts to further inhibit keloid development by inactivating the type I receptor [15]. Furthermore, Qu positively impacts the re-epithelisation process because it can inhibit interleukin-4 (IL-4) and IL-13 [8]. Although both GA and Qu are highly beneficial for preventing the formation of keloid scars and wound healing, some shortcomings may limit the therapeutic applications of oral administration. GA is a phenolic acid compound with a simple structure, containing only one benzene ring, which leads to poor stability and rapid decomposition; this is a major challenge in bioavailability [12]. However, Qu is a highly hydrophobic compound, which limits its applications in the biomedical field [16]. To overcome the poor in vivo stability and low bioavailability of GA and Qu, topical delivery has been developed as an advanced alternative strategy in recent years; however, the stratum corneum prevents ingress of microorganisms and other exogenous compounds, including many drugs. Owing to their biological nature, only molecules of less than 500 Da and a lipophilic range of log 1–3 can penetrate the stratum corneum for topical transdermal delivery [17]. Therefore, the drug can only be delivered on the skin surface and cannot effectively penetrate the lesion. To overcome this dilemma, microneedle (MN)-mediated transdermal delivery of various drugs or nanoparticles has attracted attention because MNs can directly transport the drug into the subcutaneous tissue to avoid the pain associated with hypodermic injections [18,19]. In addition, MNs equipped with several needle tips can create unobstructed microchannels to allow the drug-loaded vehicle to be transported into subcutaneous tissue more easily and evenly, which significantly improves the efficiency of transdermal drug delivery [20,21].

Drug-carrying nanoparticles stably transport the drug into the damaged site through different routes, such as orally and intravenously [22]. To control drug release and enhance therapeutic efficacy, nanotechnology (such as the use of liposomes, polymeric nanoparticles, vesicular systems, and conjugate-based delivery systems) has been introduced as a topical route, which can improve chemical stability and bioavailability and prolong the release of drugs [23,24,25]. Gelatin is a natural polymer that could be obtained from the hydrolysis of collagen extracted from the skin of porcine or bovine. The ideal microneedle must have advanced functionalization features including strong mechanical for inserting through stratum corneum, biocompatibility, biodegradability, not causing cross contamination, simple fabrication process, and high repeatability. The gelatin contains a large number of functional side groups that can control the appropriate mechanical properties via chemical crosslink, and have biocompatible and biodegradable features, making gelatin be considered as an ideal material to prepare an MN patch. Furthermore, gelatin contains a huge number of Arg-Gly-Asp peptides (RGD) that can be used as cellular binding sites to enhance the affinity between cell and carrier. In addition, the abundant functional groups in the backbones of gelatin make it chemically conjugated with various organic molecules to form an amphiphilic gelatin to encapsulate hydrophobic drug.

**Figure 1:**
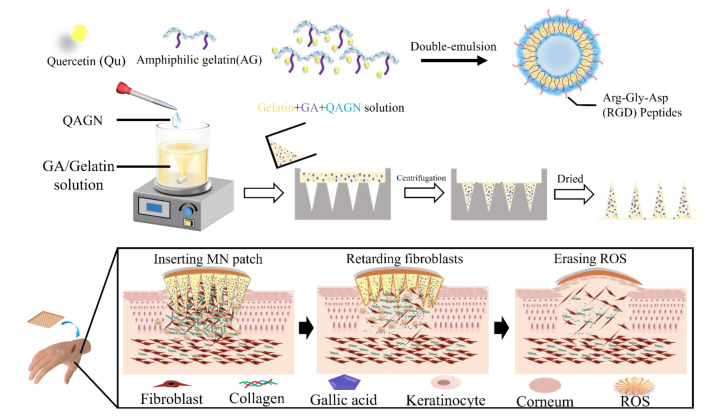
Schematic illustration of the preparation of the quercetin (Qu)-loaded amphiphilic gelatin nanocarrier and process of drug release. The prevention of keloid scars by controlling transdermal dual-drug release of Qu and GA via modulating heterogeneous gelatin-structured composite microneedles.

As is well-known, the inherent nature of MNs tends to facilitate the delivery of hydrophilic compounds. However, quercetin is a hydrophobic compound that could not be well-dissolved into gelatin solution during the fabrication process of the MN patch. In order to deliver the hydrophobic compounds through MNs, a new strategy was required to overcome the barrier by using an amphiphilic gelatin to encapsulate hydrophobic compounds such as quercetin. The MNs with both hydrophilic gallic acid and quercetin-loaded nanocarrier could stably deliver the dual drugs into the lesion site at different release stages to treat the keloid scar. In this study, we designed a heterogeneous gelatin-structured composite MN to perform transdermal dual-drug release of GA and Qu and achieve combinatory therapeutic effects, as shown in Figure 1. Specifically, amphiphilic gelatin (AG) was used to encapsulate hydrophobic Qu by forming a W/O/W drug nanocarrier via double emulsification, without additional surfactants. The RGD on the gelation can be used as cellular binding sites to enhance the affinity between cell and carrier. The hydrophilic surface of the Qu-loaded amphiphilic gelatin nanocarrier (QAGN) can be homogeneously dispersed in the gelatin of the MN which contains GA to enhance the efficiency of transdermal absorption. By controlling the release of GA and Qu, the composite MN with the nanocarrier can provide an additive effect between GA and Qu to achieve combinatory therapeutic efficacy and prevent keloid scar formation.

## 2. Materials and Methods

### 2.1. Materials

Gelatin from porcine skin, type A (300 bloom, MW: 50~100 k); gelatin from bovine skin, type B (~225 bloom, MW: 40~50 k); gallic acid with commercial reference G7384, having 97.5–102.5% (titration); 1-ethyl-3-(3-dimethyl aminopropyl) carbodiimide (EDC); N-hydroxysuccinimide (NHS); and 3-(4,5-Dimethylthiazol-2-yl)-2,5-diphenyltetrazolium bromide (MTT) were purchased from Sigma (St. Louis, MO, USA). Quercetin dihydrate and dimethyl sulfoxide (DMSO) were obtained from Merck (Darmstadt, Germany). High-glucose Dulbecco’s modified eagle medium (HG-DMEM), foetal bovine serum, trypsin-EDTA and antibiotics (penicillin/streptomycin, 200 U/mL) were purchased from GIBCO (Grand Island, NY, USA). The total RNA purification mini kit was from FavorPrep, and the iScriptTM cDNA Synthesis Kit was from Bio-Rad (Hercules, CA, USA). FastStart Universal SYBR Green Master (ROX) was purchased from Roche (Mannheim, Germany). Polydimethylsiloxane (PDMS) SYLGARD^®^ 184 was obtained from Dow Corning. Purified water (18.2 MΩ cm at 25 °C) was from Direct-Q1 ultra-pure water system, Millipore, Billerica, MA, USA. All other chemicals and solvents were of analytical grade and were used without further purification.

### 2.2. Synthesis of Amphiphilic Gelatin

The amphiphilic gelatin (AG) was synthesized and purified using a previously described preparation process [26]. Briefly, gelatin powder (3 g) was dissolved in 40 mL deionised water, and 1 mL of 1 N NaOH was added to the gelatin solution while stirring at 70 °C overnight. Ethanol (95%, 30 mL) and hexanoic anhydride (HA) (4 mL) were sequentially added to the solution and continuously stirred for 4 h. The reaction mixture was adjusted to pH 7.4 and dialyzed against ethanol for two days. AG was dried at 60 °C and ground into powder using a grinding machine.

### 2.3. Preparation of Quercetin-Loaded Amphiphilic Gelatin Nanocarrier

The QAGNs were prepared using the double emulsion method (W/O/W) in accordance with a previously described protocol, with minor modifications [26]. Briefly, AG (0.1 g) was dissolved in 4 mL of 0.05 N NaOH at 70 °C, and Qu was added at concentrations of 0.5 mg/mL, 1 mg/mL, 1.5 mg/mL, and 2 mg/mL. A mixture of 0.6 mL AG solution and 1 mL Qu solution was emulsified for 1 min to obtain a water-in-oil suspension for the first emulsion; subsequently, 2.4 mL of AG solution was added to the mixture, and the secondary emulsion was processed for 1.5 min. The mixture was stirred and heated to 60 °C for 3 h to completely remove the acetone. Finally, the products were centrifuged at 7500× *g* for 10 min to remove the precipitants with unloaded Qu. The particle suspension was repeated by centrifugation at 12,000× *g* for 15 min at 4 °C (Eppendorf Centrifuge 5810R, equipped with a rotor F-34-6-38). The particle precipitate was washed three times with deionised water. Prior to centrifugation, the particles were suspended via vigorous stirring using a vortex and a sonication bath for 5 min. The samples were washed three times with deionised water. Subsequently, the supernatant was freeze-dried at −80 °C and QAGNs were obtained in the form of dried powder for subsequent experiments.

### 2.4. Characterization of QAGNs

The morphology of the QAGNs was observed using scanning electron microscopy (SEM, SU8000, HITACHI, Tokyo, Japan). The particle size of QAGNs was measured using dynamic light scattering (DLS, Beckman Coulter Delsa™ Nano C particle analyser, Beckman Coulter, Inc., Brea, CA, USA). Samples were prepared as previously described. Briefly, the particle size was determined using DLS equipped with Dual 30 mW laser diodes. The data were analysed using the CONTIN program (DelsaNano UI software version 3.73/2.30, Beckman Coulter, Inc., Brea, CA, USA).

### 2.5. Encapsulation Efficiency and Loading Capacity

To measure the encapsulation efficiency (EE) and loading capacity (LC) of the QAGNs, the samples were subjected to centrifugation at 7500× *g* to remove unencapsulated Qu from the QAGN solution. In this process, precipitated unencapsulated Qu was dissolved in methanol to measure the absorbance of Qu at 415 nm using a UV-VIS spectrophotometer (Spectrostar Nano, BMG Labtech, Offenburg, Germany). The concentration was calculated by referring to the standard curve established from the standard Qu solutions in methanol. EE and LC were calculated using the following equations:

$$\mathrm{EE}\left(\%\right)=\frac{\mathrm{Total}\;\mathrm{amount}\;\mathrm{of}\;\mathrm{Qu}\;\left(\mathrm{mg}\right)-\mathrm{amount}\;\mathrm{of}\;\mathrm{Qu}\;\mathrm{in}\;\mathrm{precipitate}\left(\mathrm{mg}\right)}{\mathrm{Total}\;\mathrm{amount}\;\mathrm{of}\;\mathrm{Qu}}$$

$$\mathrm{LC}\left(\mathrm{mg}/g\right)=\frac{\mathrm{Total}\;\mathrm{amount}\;\mathrm{of}\;\mathrm{Qu}\left(\mathrm{mg}\right)-\mathrm{amount}\;\mathrm{of}\;\mathrm{Qu}\;\mathrm{in}\;\mathrm{precipitate}\left(\mathrm{mg}\right)}{\mathrm{Total}\;\mathrm{amount}\;\mathrm{of}\;\mathrm{QAGN}\left(g\right)}$$

### 2.6. Qu Release Study

To monitor the QAGN release of Qu over time, the amount of Qu released from the QAGN was determined via a spectrophotometric assay. The percentage of Qu released was calculated as the total amount of Qu loaded into the samples. Briefly, 10 mg of lyophilised QAGNs was added to 40 mL phosphate-buffered saline (PBS, pH = 7.4), and the solution was subjected to ultrasonication until adequate dispersion and maintained with gentle stirring at 37 °C. At selected times, the samples were collected in Eppendorf tubes and centrifuged (12,000× *g* for 20 min at room temperature), and the supernatants of Qu were determined spectrophotometrically at 415 nm. The cumulative release (%) was calculated from the total Qu loaded into the QAGN and plotted against time.

### 2.7. Synthesis of MN Arrays Patch

First, type B gelatin powder was added to deionised water to prepare different concentrations of gelatin solution, including 10%, 15%, and 20% *w*/*v*, heated to 50 °C, and stirred until the gelatin was completely dissolved. EDC/NHS (0.0575 M EDC and 0.0115 M NHS) was added to the gelatin solution and continuously stirred for 8 h for crosslinking. Next, the prepared solution was poured into polydimethylsiloxane (PDMS) moulds and placed in 50 mL tubes containing PDMS inserts. After that, the tubes were centrifuged in a swinging bucket rotor at 4000 rpm for 30 min at 40 °C to fill the cavities of the PDMS mould and form the tips of the MNs. Then, these moulds were dried in an oven at 4 °C overnight. Drug (150 μM GA)-loaded (10 mg QAGN) mixture of GA and QAGN MN patches were fabricated using the same production process. The MN patches were stored in a humidity control cabinet until further use.

### 2.8. Mechanical Test of MN Patch

A universal testing machine (AGS-500NX; Shimadzu Corporation, Kyoto, Japan) was used to determine the mechanical strength of the MN array. Briefly, the MN patch was laid on the flat rigid surface of the stainless-steel base stage, and the pressure force was recorded by the moving sensor at a constant speed of 0.02 mm/min. The initial distance from the tips of the MN arrays to the mount was set as 8 mm. Once the tip of the MN array touched the moving sensor, the testing machine recorded the force required to move the mount as a function of the MN displacement.

### 2.9. In Vitro Penetration Capability of MN Patch

To determine the in vitro skin insertion capability, the drug-loaded BMN patch was inserted into porcine cadaver skin in a vertical pressure direction for 1 min. After insertion, the insertion site was immediately stained with 0.4% trypan blue dye for 3 min to mark the penetration sites of stratum corneum perforation. Subsequently, the skin was washed to remove residual dye from the surface, and skin sections were used to evaluate MN penetration ability. The penetration ratio was calculated by dividing the number of blue dots on the skin after insertion for each MN patch, and the insertion depth was defined as the average penetration of needles that could be successfully inserted into the skin from the five MN arrays. Histological sections of the MN penetration sites were excised from the skin using a scalpel. Skin samples were fixed in 4% paraformaldehyde, embedded in paraffin, sliced into 5 μm sections, and stained with haematoxylin and eosin (H&E).

### 2.10. In Vitro Drug Permeation Study

The MN patch containing QAGN or GA was inserted on the surface of Parafilm M^®^, which was fixed onto 5 mL PBS and incubated in a water bath under stirring at 200 rpm and 32 °C. The solution was periodically collected to determine the concentrations of QE and GA using an individual standard curve with an ELISA microplate reader. The released GA was determined using the Folin–Ciocalteu reagent, and 100 µL of each sample solution was mixed with 500 µL of water and then with 100 µL of the Folin–Ciocalteu reagent and allowed to stand for 6 min. Subsequently, 1 mL of 7% sodium carbonate and 500 µL of distilled water were added to the reaction mixture. Absorbance was recorded after 90 min at a wavelength of 760 nm. The Qu release study was performed for MN patches containing QAGN following a similar experimental method. The solution was collected and centrifuged to remove the supernatant, and the precipitate containing Qu was dissolved in pure ethanol. The absorbance of released Qu was determined at a wavelength of 410 nm, and the concentration of released Qu was calculated based on the Qu standard curve.

### 2.11. Cell Proliferation and Viability Assay of L929 Fibroblasts

L929 mouse fibroblasts were obtained from the Bioresource Collection and Research Center (BCRC, Hsinchu, Taiwan). L929 fibroblast cells were cultured in high-glucose Dulbecco’s modified Eagle medium supplemented with 10% bovine serum and antibiotics and incubated at 37 °C in a humidified 5% CO_2_ atmosphere. Adequate fibroblast cells were harvested from 75T culture flasks and seeded in 6-well plates at a density of 5 × 10^4^ cells/well. Fibroblast cells were treated with various conditions of the experimental group (including blank, pure MN, GA + MN, QAGN + MN, and GA + QAGN + MN) for 1, 3, 5, and 7 days to evaluate the influence of fibroblast proliferation. At a predetermined time, the early cultured medium was replaced with a serum-free medium containing 3-(4,5-dimethylthiazol-2-yl)-2,5-diphenyltetrazolium bromide (0.5 mg/mL) for 3 h prior to centrifugation at 12,000 rpm for 5 min. After discarding the supernatants, the formazan products were dissolved in 250 μL dimethyl sulfoxide by vortexing. The absorbance of samples was measured at 570 nm using a microplate reader (Spectrostar Nano, BMG Labtech, Offenburg, Germany).

### 2.12. Aniline Blue Staining

Fibroblast morphology and collagen quantity were assessed via Masson trichrome staining. Briefly, the culture medium was removed, and the cells were washed with PBS and fixed with methanol at room temperature. Bouin’s solution (HT 10132; Sigma-Aldrich, St. Louis, MO, USA) was added to the plate for 15 min. Subsequently, cells were washed with PBS for 5 min. Next, the cells were stained with Weigert’s haematoxylin for 5 min and washed again with PBS for 5 min. The cells were then stained with aniline blue for 5 min and fixed in 1% acetic acid for 2 min. Finally, the cells were rinsed with distilled water, dehydrated, and mounted for microscopic observation.

### 2.13. Measurement of Reactive Oxygen Species Generation

L929 fibroblasts were seeded on a 96-well plate (black) at a density of 1.0 × 10^5^ cells/mL overnight. Following the manufacturer’s instructions, we added the fluorescent probe 2′-7′-dichlorodihydrofluorescein diacetate (DCF-DA) (ab113851, Abcam, Cambridge, UK) to the culturing plate for 45 min at 37 °C in the dark to measure cell oxidation. The original medium was removed, washed with PBS, and cells were cultured and treated with 100 µm tert-butyl hydroperoxide (TBHP) culture medium (90 µL medium: 10 µL TBHP) for 24 h to induce the generation of reactive oxygen species (ROS). Subsequently, the MN patches were fixed on the cell plate for 24 h, and cells were labelled with 100 µL of 20 µM DCF-DA for each well for 45 min at 37 °C in the dark. Fluorescence (485 nm excitation/535 nm emission) was measured using an ELISA plate reader. The percentage of the control for ROS was calculated as follows:

$$\mathrm{ROS}\left(\%\;\mathrm{of}\;\mathrm{control}\right)=\frac{\left(\mathrm{Fluorescence}\;\mathrm{of}\;\mathrm{MN}\;\mathrm{patches}\;\mathrm{treated}\;\mathrm{cells}-\mathrm{Fluorescence}\;\mathrm{of}\;\mathrm{blank}\right)}{\left(\mathrm{Fluorescence}\;\mathrm{of}\;\mathrm{TBHP}\;\mathrm{treated}\;\mathrm{cells}-\mathrm{Fluorescence}\;\mathrm{of}\;\mathrm{blank}\right)}\times 100\%$$

### 2.14. Real-Time Quantitative Polymerase Chain Reaction

The polymerase chain reaction (PCR) procedure was described in our previous study [27]. Briefly, total RNA was extracted using a FavorPrep Tissue Total RNA Mini Kit, following the manufacturer’s protocol. RNA was reverse-transcribed to cDNA using the iScriptTM cDNA Synthesis Kit. cDNA was amplified using SYBR green PCR reagents and real-time quantitative PCR (*q*RT-PCR) was conducted using the StepOneTM real-time PCR system (Applied Biosystems, Waltham, MA, USA). Primer sequences for the target genes are listed in Table S1 (Supplementary Materials). GAPDH was used as a housekeeping gene. All reactions were performed in duplicate, and relative expression levels were calculated using the 2^−ΔΔ*C*T^ method.

### 2.15. Statistical Analysis

All the data were expressed as mean ± standard deviation (SD). The data were compared by one-way analysis of variance (ANOVA) to evaluate differences among the groups. A difference with *p* < 0.05 was considered statistically significant. *p* < 0.05 (*); *p* < 0.01 (**); and *p* < 0.001 (***). Statistical analyses were performed with GraphPad Prism 8.0 (GraphPad Software, Inc., San Diego, CA, USA).

## 3. Results

### 3.1. Preparation and Characterization of QAGNs

**Figure 2:**
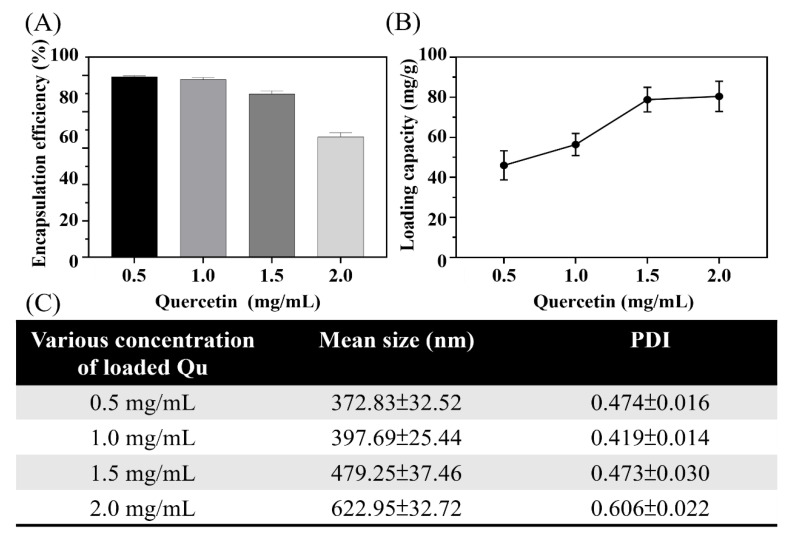
(**A**) Encapsulation efficiency and (**B**) loading capacity of Qu-encapsulated nanoparticles with various concentrations (0.5, 1, 1.5, 2 mg/mL). (**C**) Particle size distribution and polydispersity index (PDI) of Qu-encapsulated QAGN with various concentrations (0.5–2 mg/mL). Data are shown as mean ± SD (*n* = 5).

Qu is a flavonoid compound which exists in numerous natural plants and has many positive effects in preventing various diseases, with multiple pharmacological properties, including antioxidant, anti-inflammatory, and anticancer properties. However, the poor water solubility of Qu limits its bioavailability so an amphiphilic (AG) was synthesized to encapsulate the hydrophobic Qu and further improve its bioavailability. The synthesis and analysis of AG were described as below. First, HA was grafted onto gelatin to replace the Arg molecule. The proton nuclear magnetic resonance spectra in Figure S1 show different chemical shift signals between primitive gelatin and HA-grafted gelatin, labelled A (2–2.3 ppm) and B (0.9 ppm), respectively. In addition, the amino group of the primer gelatin (2.8 ppm) of the original site was substituted by a hexanoyl group. Thereafter, AG with different concentrations (0.5, 1, 1.5, and 2 mg/mL) of Qu was used to synthesize the QAGNs via a simple double-emulsion process. After the acetone was completely volatilised and centrifuged, the QAGNs were obtained (Figure S2). The morphology and particle sizes of the QAGNs were investigated by SEM and DLS. The morphology of the QAGNs with concentrations of Qu ranging from 0.5 mg/mL to 2.0 mg/mL presented a spherical shape with a diameter varying from 400 nm to 650 nm (Figure S3A–D). The particle sizes were measured via DLS as 372.83 ± 32.52, 397.69 ± 25.44, 479.25 ± 37.46, and 622.95 ± 32.72 nm with increasing Qu loading (Figure 2C), which were consistent with observations from the SEM images. In addition, as the Qu concentration increased, the polydispersity index value (PDI) of QAGN increased from 0.419 to 0.606. The EE and LC were also measured. As shown in Figure 2A,B, the EE was measured at over 90% in QAGN with Qu concentrations of 0.5, 1.0, and 1.5 mg/mL; however, the EE significantly decreased to 66.25% with 2 mg/mL of Qu, which may be attributed to the exceeded supersaturation levels when adding Qu to the AG dispersions. In addition, the LC curve did not exhibit a significant difference between 1.5 mg/mL and 2 mg/mL. Therefore, 1.5 mg/mL Qu encapsulated in QAGN was used for subsequent experiments. The in vitro release of Qu (Figure S4) suggested an initial burst release after the first day (14.15%, 16.83%, 24.55%, and 27.22% for QAGN with Qu concentrations of 0.5, 1.0, 1.5, and 2.0 mg/mL, respectively), followed by slow release. The burst release may partially result from loosely attached Qu particles at the surface of the QAGNs that were easily dissociated. Subsequently, it presented a sustained release independent of the Qu loading concentration. The maximum released amounts reached 34.33%, 42.72%, 57.55%, and 53.00% with 0.5–2.0 mg/mL Qu loading QAGN on day 7.

### 3.2. Preparation and Characterization of MNs

**Figure 3:**
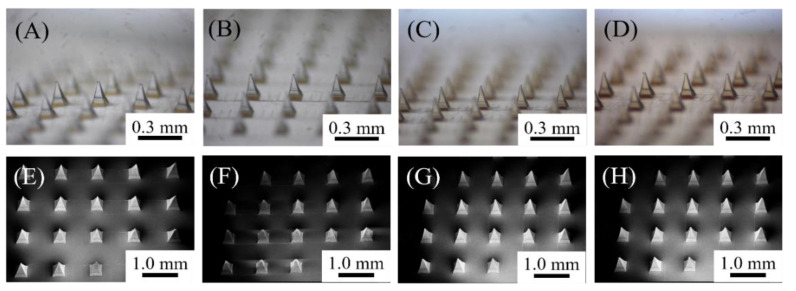
Light microscope photograph of MN patches with various loading conditions: (**A**) pure Gel, (**B**) GA-loaded, (**C**) QAGN-loaded, and (**D**) GA + QAGN-loaded MN patch. The corresponding SEM images of MN patches with various loading conditions are shown in (**E**–**H**).

Heterogeneous composite gelatin-structured MNs were fabricated using a simple centrifugal casting process. The MN patch consisted of 81 (9 × 9) needles. The base width and height of both the MN and the supporting structure were 300 µm and 600 µm, respectively. During the fabrication of the MN patch, GA, QAGN, or both were added to the gelatin hydrogel (Gel) until a homogeneous mixture was obtained through gelatin crosslinking with EDC/NHS. Thereafter, the heterogeneous gelatin hydrogel was loaded onto a female mould to prepare MN patches by centrifugation. As shown in the microscope photomicrograph (Figure 3A–D for pure Gel, GA-, QAGN-, and GA + QAGN-loaded MN patches, respectively), a needle structure with a layered structure was formed in various groups after demoulding, as expected. In addition, detailed microscopic observations of the MN patch were performed using SEM. As shown in the SEM images (Figure 3E–H for pure Gel, GA-, QAGN-, and GA + QAGN-loaded MN patches, respectively), the pyramidal needles were regularly arranged on the base substrate without any fractures or breaks after the hydrogel MNs had dried.

### 3.3. MN Functionality Testing

Gelatin is a highly hydrophilic natural polymer that easily absorbs the moisture of subcutaneous tissue after insertion into the skin surface, which makes the needle easily deformed and complicates the preservation of sharpness and stiffness. Therefore, we prepared MNs with various concentrations of gelatin to evaluate the optimal gelatin concentration via mechanical strength analysis, an insertion ratio test, and hygroscopicity measurements. The mechanical strength of the MN patch with 20% Gel reached 5.5 N, indicating that the 20% Gel concentration maintained a stable insertion ability for loading GA and QAGN (Figure S5A). Furthermore, only the MN patch composed of 20% Gel could achieve the thickest insertion depth (508 μm) and higher hole-created ratio (62.5%), as shown in Figure S5B, indicating that the concentration of Gel must be at least 20% to offer a more stable penetration ability for insertion into the stratum corneum. To further confirm whether GA and QAGN loading would influence the mechanical properties of the MN and reduce skin insertion, various MN patches were prepared to test their insertion ability. Since the biological samples have some unstable situations such as heterogeneity and low reproducibility, some literature reports proposed other alternative approaches to overcome the limitations of in vitro MN testing by replacing biological tissues with synthetic membranes such as Gantrez^®^, Parafilm M^®^ and Deka^®^ [28,29,30]. Among these synthetic films, Parafilm M^®^ has the physical properties closest to porcine skin and can substitute for biological samples. Therefore, we used Parafilm M^®^ as the model skin barrier to conduct the in vitro experiments.

**Figure 4:**
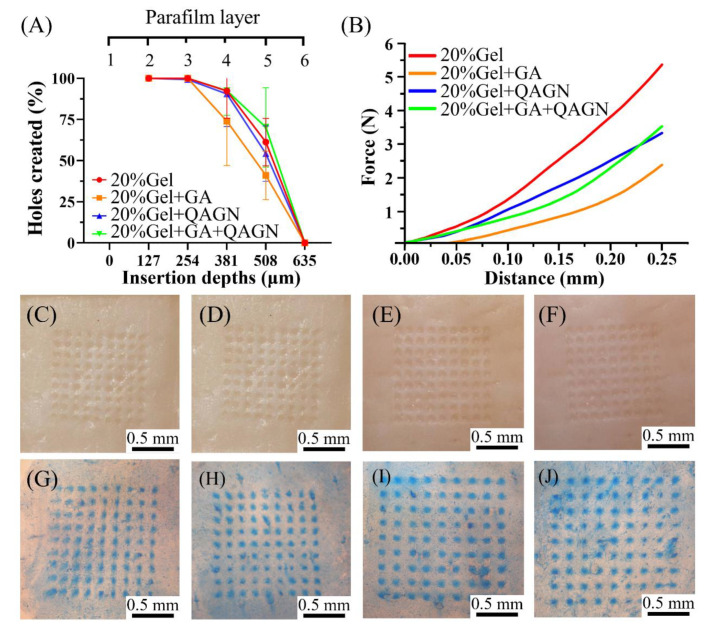
Evolution of MN patches’ insertion ability. (**A**) Mechanical properties analysis; (**B**) Parafilm insertion simulations. The porcine skin insertion photographs represent (**C**) pure Gel, (**D**) GA-loaded, (**E**) QAGN-loaded, and (**F**) GA + QAGN-loaded MN patches. The trypan blue staining for labelling the porcine skin insertion locations represents (**G**) pure Gel, (**H**) GA-loaded, (**I**) QAGN-loaded, and (**J**) GA + QAGN-loaded MN patches.

**Figure 5:**
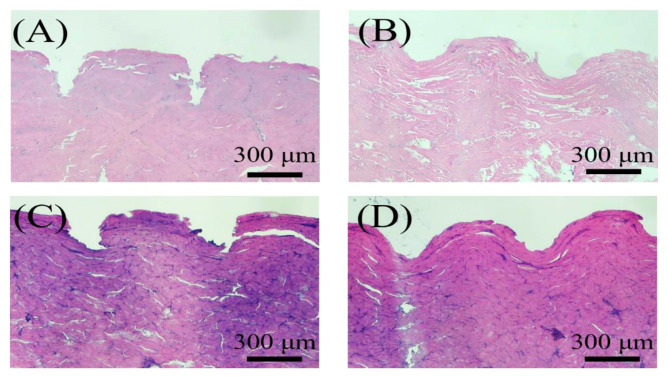
Images of H&E-stained skin sections after the application of (**A**) pure Gel, (**B**) GA-loaded, (**C**) QAGN-loaded, and (**D**) GA + QAGN-loaded MNs.

As shown in Figure 4A, all MN patches were successfully inserted to a depth of 506 µm. Mechanical testing (Figure 4B) indicated that the unloaded MN patch had the strongest mechanical properties, and GA-loaded MN patches had lower mechanical strength (2 N). However, the strength was still much greater than the force required for each needle to penetrate the skin (0.1 N) [31]. Furthermore, the pure Gel, GA-loaded, QAGN-loaded, and GA + QAGN-loaded MN patches were further inserted into the porcine cadaver skin, and the marking sites in the porcine skin insertion photographs were observed (Figure 4C–F). The corresponding MN insertion sites were further stained with blue dye to calculate the penetration ratio of all groups up to almost 100% (Figure 4G–J). Based on the histological sections shown in Figure 5, the insertion depths were measured at 470, 300, 340, and 420 μm for pure Gel, GA-loaded, QAGN-loaded and GA + QAGN-loaded MN patch, respectively. The results showed no significant difference between the QAGN and GA + QAGN groups. However, the unloading drug (empty) and GA groups had lower mechanical strength and insertion thickness compared with the above groups, which was perhaps attributed to the moisture absorption of Gel.

### 3.4. In Vitro Drug Release Profile of Dual-Drug-Loaded MNs

**Figure 6:**
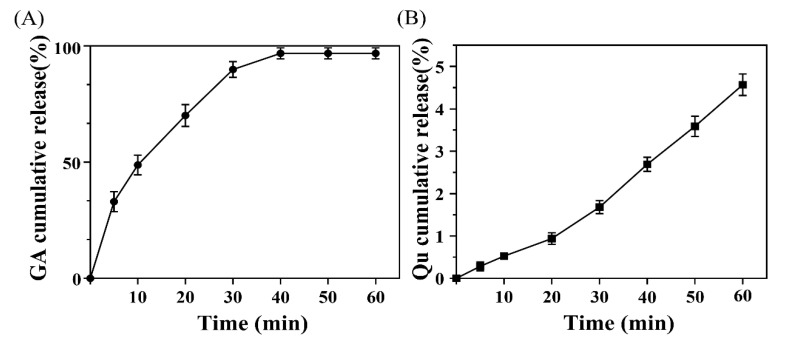
Drug permeation profiles of MN patches: (**A**) GA release curve; (**B**) Qu release curve (mean ± SD, *n* = 5).

To investigate the dual-drug release from the heterogeneous composite gelatin-structured MNs, the MN patches were inserted onto the surface of the parafilm, which was fixed onto PBS to monitor drug release. Figure 6A shows that GA was rapidly released within 40 min after contact with the PBS-simulated interstitial fluid and completely released from the MN patches after 40 min. In contrast, Qu in QAGNs showed sustained and stable release over time in Figure 6B. At 40 min, only approximately 3% was released, which was attributed to the encapsulation of QA in the QAGNs.

### 3.5. Cell Proliferation and Viability Assay of L929 Fibroblasts

**Figure 7:**
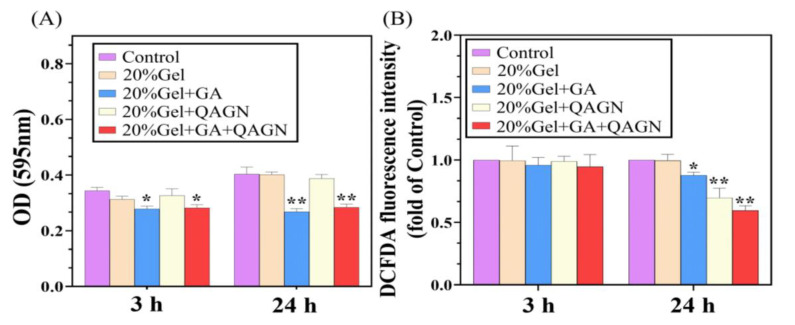
MN patches for in vitro biological activity analysis. (**A**) Cell proliferation assessment: chondrocytes were co-cultured with various loaded MN patches for 3 and 24 h (data are presented as mean ± SD, * *p* < 0.05, ** *p* < 0.01); (**B**) ROS relative content analysis (mean ± SD, *n* = 5).

To evaluate the effect of released GA or Qu on the cell proliferation and viability in this study, fibroblasts were cultured at a high density to simulate the keloid scar formation process, and the cells were treated with various loaded MN patches. As shown in Figure 7A, after incubation with GA or GA + QAGN-loaded MN patches for various times, cell viability decreased significantly compared to the control group. In contrast, the gelatin (Gel) and QAGN groups did not appear obviously different from the control, indicating that they did not adversely affect the metabolic activity of fibroblasts. However, the proliferation of fibroblasts in both the GA and GA + QAGN groups declined after 3 h and significantly decreased after 24 h of culture, indicating that GA could retard the proliferation of fibroblasts over time. In addition, Qu is considered to have excellent antioxidant molecules for preventing keloid scar formation; therefore, ROS generation was necessary to evaluate the efficacy of preventing keloid scar formation in this study. Figure 7B shows that ROS was slightly downregulated, with no significant difference in all groups at 3 h but apparent inhibition of ROS generation in QAGN and GA + QAGN at 24 h.

### 3.6. Aniline Blue Staining of L929 Fibroblasts

**Figure 8:**
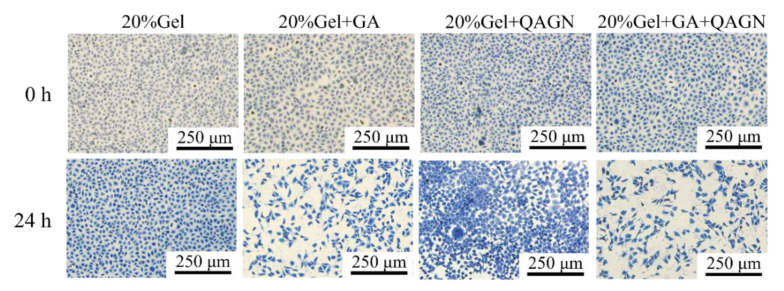
Collagen content staining with various loaded MN patches for 24 h.

Another important indicator of keloid scar formation is excessive collagen deposition. Aniline blue was used to stain collagen production to evaluate the inhibitory effect of dual-drug release in the heterogeneous gelatin-structured composite MNs on collagen. The results in Figure 8 indicate that the fibroblasts continuously and rapidly proliferated to form high-density collagen in the control group. In contrast, the collagen was inhibited in the other groups. The deposited collagen exhibited the most dramatic reduction in GA-treated MN patches, especially in the GA + QAGN group.

### 3.7. qRT-PCR

**Figure 9:**
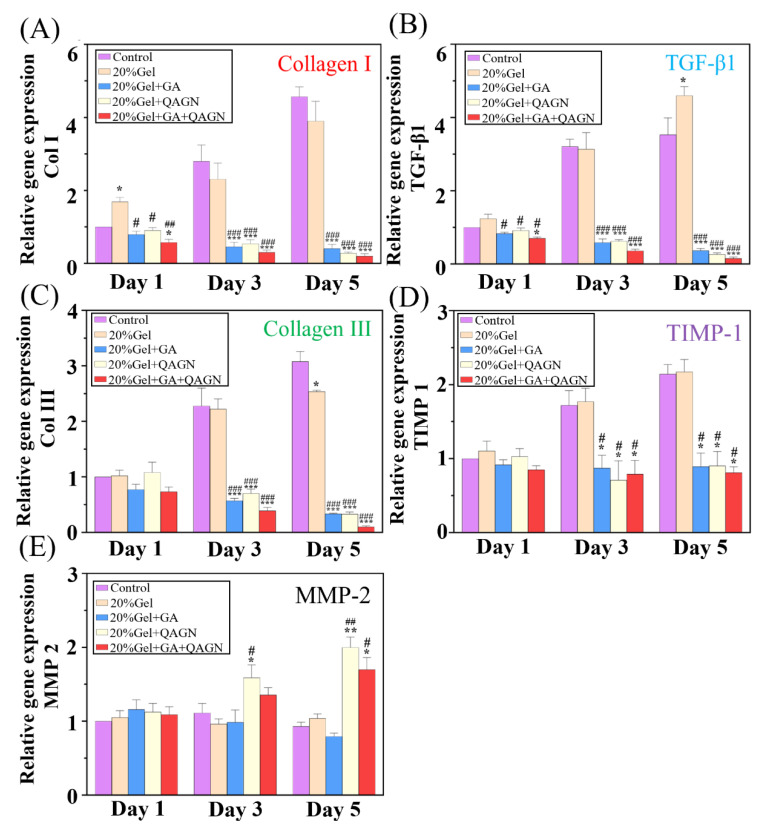
Relative expression levels of keloid scar and genes for extracellular-matrix-degrading metalloproteinases, including (**A**) Col I, (**B**) TGF-β1, (**C**) Col III, (**D**) TIMP-1, and (**E**) MMP-2 analysed to detect the influence of QAGN and GA on fibroblasts. Data are represented mean ± SD, * compared with the control group. * *p* < 0.05, ** *p* < 0.01, *** *p* < 0.001, and ^#^ compared with the gelatin group ^#^ *p* < 0.05, ^##^ *p* < 0.01, ^###^ *p* < 0.001.

Col I, Col III, and TGF-β1 have been widely considered as vital index genes of keloid scars. Therefore, the expression of these genes was detected using *q*RT-PCR to investigate fibroblasts after treatment with the MN patches. Figure 9A–C show that the relative expression of Col I and TGF-β1 was downregulated in the MN patches loaded with GA, QAGN, and GA + QAGN groups on day 1 compared with that of the control group. The expression of Col I, Col III, and TGF-β1 was progressively decreased with treatment time. As Qu was gradually released from QAGN, a relative inhibitory effect was observed on day 5. In addition, scar formation also depended on the balance between cellular matrix proteolytic factors, including metallopeptidase inhibitor 1 (TIMP-1) and matrix metalloproteinase 2 (MMP-2). It was found that compared to that in the control group, the expression of TIMP-1 in fibroblasts was downregulated in the GA, QAGN, and GA + QAGN groups, with the latter showing the most significant difference. In contrast, the MMP-2 relative expression level was increased in the QAGN and GA + QAGN groups over time.

## 4. Discussion

Keloid scarring is a chronic skin disease characterised by abnormal fibroblast proliferation and excessive collagen deposition in scar skin. Therefore, inhibition of fibroblast proliferation is currently the main strategy against keloid scar formation. To fully inhibit keloids, the MN patches were designed to control transdermal dual-drug (GA and Qu) release into lesion sites in the skin to prevent keloid scars. Gelatin with excellent biocompatibility and biodegradation was selected to prepare the hydrogel MN patches. To enhance the mechanical properties of the MN patches over a force of 2 N (Figure 4A), EDC/NHS was used to crosslink gelatin. GA is a plant polyphenol with an inhibitory effect on the proliferation of keloid scar fibroblasts. Therefore, GA was added in the gelatin to fabricate the GA-loaded MNs for benefit direct delivering into the wound skin. The result in Figure 6A showed that GA was rapidly released at an earlier stage due to the dissolution of MN to inhibit fibroblast proliferation, as demonstrated in Figure 7A. Cell viability was rapidly inhibited after GA was released in the short term (3 h) and displayed a significant downward trend after 24 h, particular in the GA and GA + QAGN groups. In contrast, the QAGN group did not present any obvious effect on the inhibition of fibroblast proliferation. Therefore, the contribution from the GA is very important in inhibiting the abnormal fibroblast proliferation. To further avoid excessive collagen deposition and prevent scar formation in the later stage, Qu was used because it is an onion extract that possesses multiple hydroxyl groups and can erase ROS [32]. However, Qu is hydrophobic and not easily dispersed in an aqueous solution for cellular uptake, restricting its bioavailability and causing a major barrier to therapeutic uses [33]. To improve the low bioavailability and poor absorption of Qu, AG nanoparticles was synthesized to encapsulate Qu. As expected, Qu in QAGN can be released slowly and continuously as shown in Figure S4. The ROS activity was significantly inhibited in the QAGN compared to GA groups after 24 h of culture, indicating the later-released Qu made a significant contribution to the inhibition of ROS. It is noted that the group of GA + QAGN can much enhance the efficacy, indicating that the combination of GA and Qu has the most significant inhibitory effect on fibroblast proliferation and ROS generation. In addition to retarding fibroblast proliferation, the regulation of additional ECM production (such as Col I and III) which was involved in collagen synthesis is also important for preventing keloid scar formation. Therefore, Col I, Col III, and TGF-β1 can be used as vital index genes for keloid scars [34]. The results in Figure 9A–C further demonstrated that the expression level of Col I, III, and TGF-β1 was decreased with the time period in the groups of MN patches with QAGN and GA + QAGN groups, indicating that the sustained Qu release could significantly inhibit the fibrosis-related gene expression of keloid scars. The inhibition effect was correlated with Qu because Qu has been reported to attenuate wound fibrosis by suppressing the TGF-β1/Smad signalling pathway and activating the PI3K/Akt signalling pathway to inhibit collagen overproduction [35]. The inhibition on collagen deposition can be further evidenced from the balance between TIMP-1 and MMP-2 which are key regulators of the ECM in Figure 9D–E. The increased MMP-2 relative expression level in the QAGN and GA + QAGN groups further supported that the MN with GA + QAGN group could reduce excessive ECM deposition and prevent keloid scarring through the combinatory effect. In this study, the preliminary in vitro results have demonstrated that the composite dual-drug microneedle system could effectively achieve the inhibition effect on the formation of keloid scars via the different release profiles of GA and Qu. However, the skin tissue structure of keloid is slightly different from that of healthy skin. Therefore, we will use an appropriate animal model to conduct an in vivo experiment, which is essential to investigate the feasibility of the composite microneedle in the treatment of keloid scars. In addition, to realize the possibility of the MN system, the depth of microneedle penetration into the epidermis, the degradation of the microneedle in vivo, the improvement in drug persistence, and the combinatory therapeutic effects of the dual drugs in epidermal tissue will be explored in the future.

## 5. Conclusions

We successfully designed a heterogeneous gelatin-structured composite MN system which modulated dual-drug release profile of GA and Qu to prevent keloid scar formation. The developed MN system can realise a combinatory effect by rapidly releasing the gallic acid due to the rapid dissolution of the MN after insertion, thereby effectively inhibiting the fibroblast proliferation and production of collagen. In addition, nanocarriers in the MNs can achieve a slow and sustained release of quercetin, thereby reducing the production of reactive oxygen species. In turn, the relative gene expression of Col I, III, and TGF-β1 was continuously downregulated to inhibit keloid scar formation for the MN with GA + QAGN group. Our preliminary results showed that the composite microneedle system shows great potential to realize the possibility of preventing keloid scar formation and in vivo experiments will be explored in the future.

## Data Availability

Not applicable.

## Supplementary Materials

The following supporting information can be downloaded at: https://www.mdpi.com/article/10.3390/polym14204436/s1, Figure S1. 1H-NMR spectrum of gelatin molecules in D2O: (A) primitive and (B) modified gelatin molecules; Figure S2. Appearance of QAGN with loading different concentrations of quercetin, in deionized water; Figure S3. Particle size and SEM image of QAGNs with various concentrations of quercetin. (A)0.5mg/ml quercetin loaded QAGNs; (B) 1mg/ml quercetin loaded QAGNs; (C)1.5mg/ml quercetin loaded QAGNs; (D) quercetin loaded QAGNs; Figure S4. Drug releasing profiles of Qu in QAGNs; Figure S5. (A) Mechanical properties analysis of composite MN patches with various concentrations of gelatin; (B) Insertion in parafilm; Table S1. Primers used in this study.
