# Supplementary material for: The Treatment of Keloid Scars via Modulating Heterogeneous Gelatin-Structured Composite Microneedles to Control Transdermal Dual-Drug Release
Source: Polymers (Basel). 2022 Oct 20;14(20):4436. doi: 10.3390/polym14204436 (PMC9607586; doi:10.3390/polym14204436)
Supplement: Supplementary file 1 [file polymers-14-04436-s001.zip › polymers-1918275-supplementary.pdf]

Supplementary data

Table S1. Primers used in this study.

Marker gene		Sequences (5'-3')	Accession No.
Gapdh	Forward	GACTCCACTCACGGCAAATTCAAC	XM_036165840
	Reverse	GTCGTGGAGTCTACTGGTGTCTTCAC	
Col I	Forward	CCCAGTGGCGGTTATGACTTCA	NM_007742
	Reverse	CACCACCCTCAAGAGCCTGAGT	
TGF-β1	Forward	GAGCCACTGCCCATCGTCTACTAC	NM_011577
	Reverse	GCTACCATGCCAACTTCTGTCTGG	
Col III	Forward	TTCATGAAGATGACCGACGA	NM_009930
	Reverse	GTCCAGTCGTAGCCCTTGAG	
TIMP-1	Forward	CCTTCTGCAACTCGGACCTGGTC	NM_001044384
	Reverse	GCTATCACTGATAGCTTCCAG	
MMP-2	Forward	GAGCATGGAGATGGATACC	NM_008610
	Reverse	GTATGGAGCGACGTCACTCC	

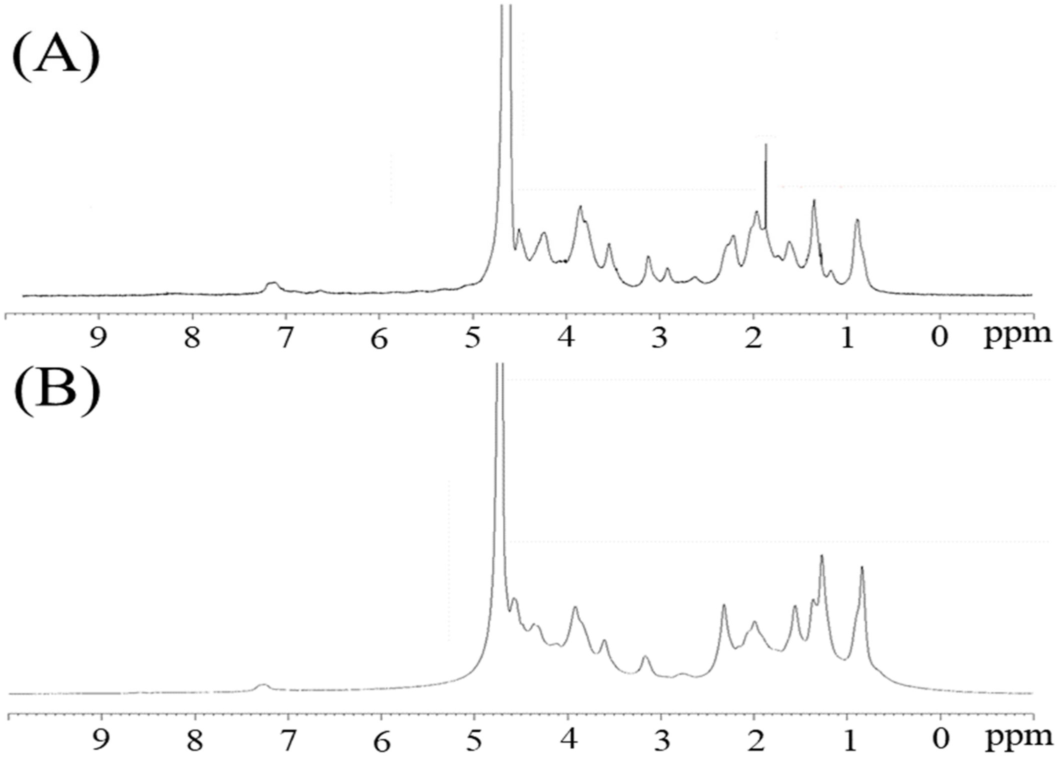

Figure S1. ^{1}H-NMR spectrum of gelatin molecules in D_2O: (A) primitive and (B) modified gelatin molecules.

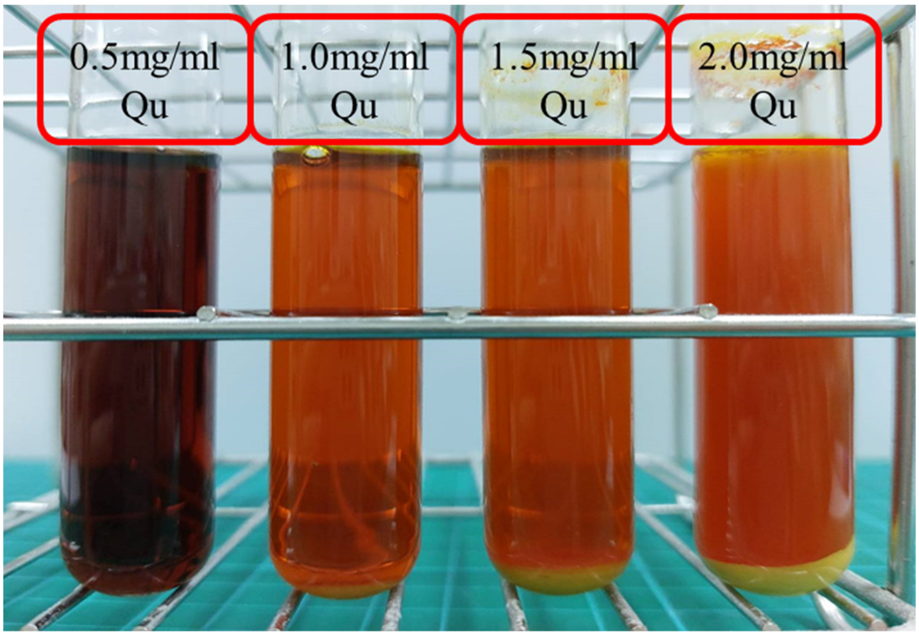

Figure S2. Appearance of QAGN with loading different concentrations of quercetin, in deionized water.

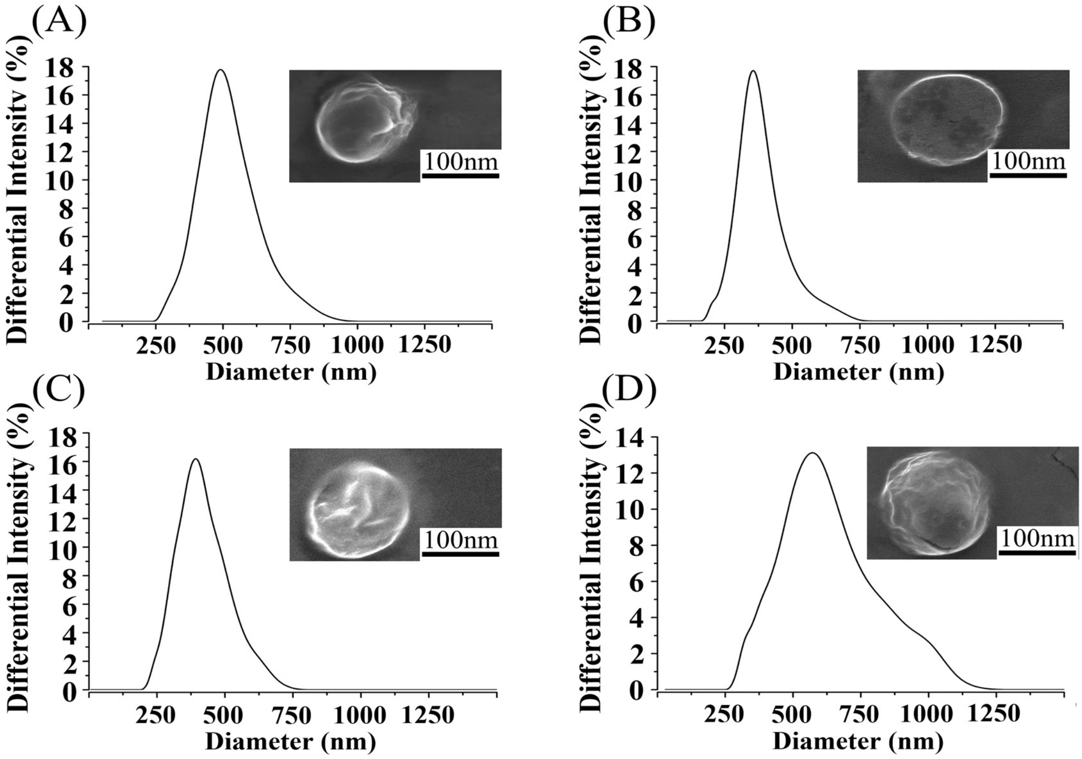

Figure S3. Particle size and SEM image of QAGNs with various concentrations of quercetin. (A)0.5mg/ml quercetin loaded QAGNs; (B)1mg/ml quercetin loaded QAGNs; (C)1.5mg/ml quercetin loaded QAGNs; (D) quercetin loaded QAGNs.

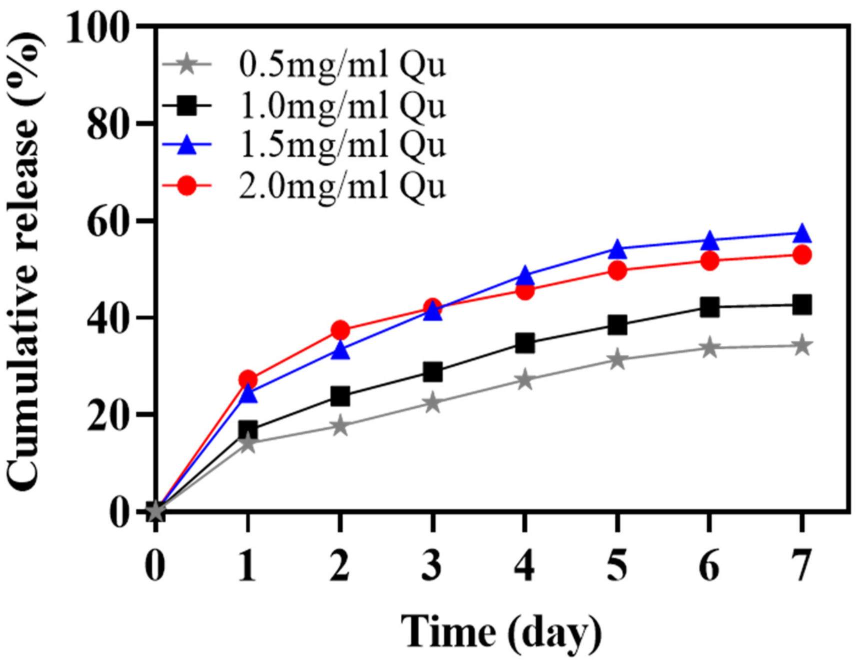

Figure S4. Drug releasing profiles of Qu in QAGNs.

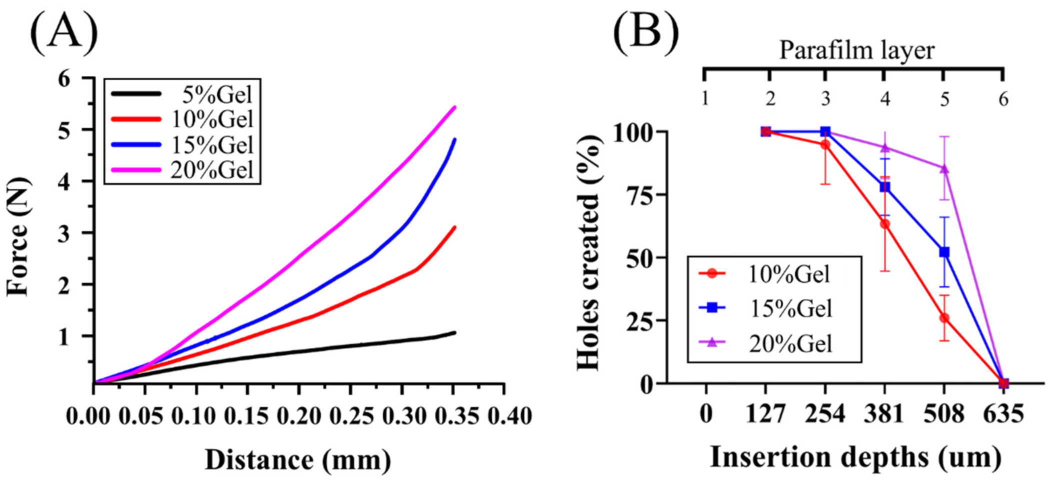

Figure S5. (A)Mechanical properties analysis of composite MN patches with various concentrations of gelatin; (B) Insertion in parafilm.
